# Analysis of the septal curvature with CMR in the paediatric population with pulmonary hypertension is a useful tool

**DOI:** 10.1186/1532-429X-14-S1-P83

**Published:** 2012-02-01

**Authors:** Bejal Pandya, Shahin Moledina, Andrea McKee, Ingram-Nieck Schulze, Vivek Muthurangu

**Affiliations:** 1 Cardiac Imaging, Institute of Cardiovascular Science, University College London, London, UK

## Background

Paediatric pulmonary hypertension is often difficult to assess non-invasively. Early detection and treatment of mild disease in high-risk populations e.g. pulmonary disease is associated with improved outcome. In such cases, echocardiographic measurement of tricuspid regurgitation is unreliable for estimation of right heart pressure. Cardiac MR imaging offers an alternative, using deformation of the interventricular septum to assess the pressure differential between the ventricles. In this study, we hypothesised that this technique offers a useful tool for both detection of pulmonary hypertension and assessment of those with established disease.

## Methods

**Figure 1:**
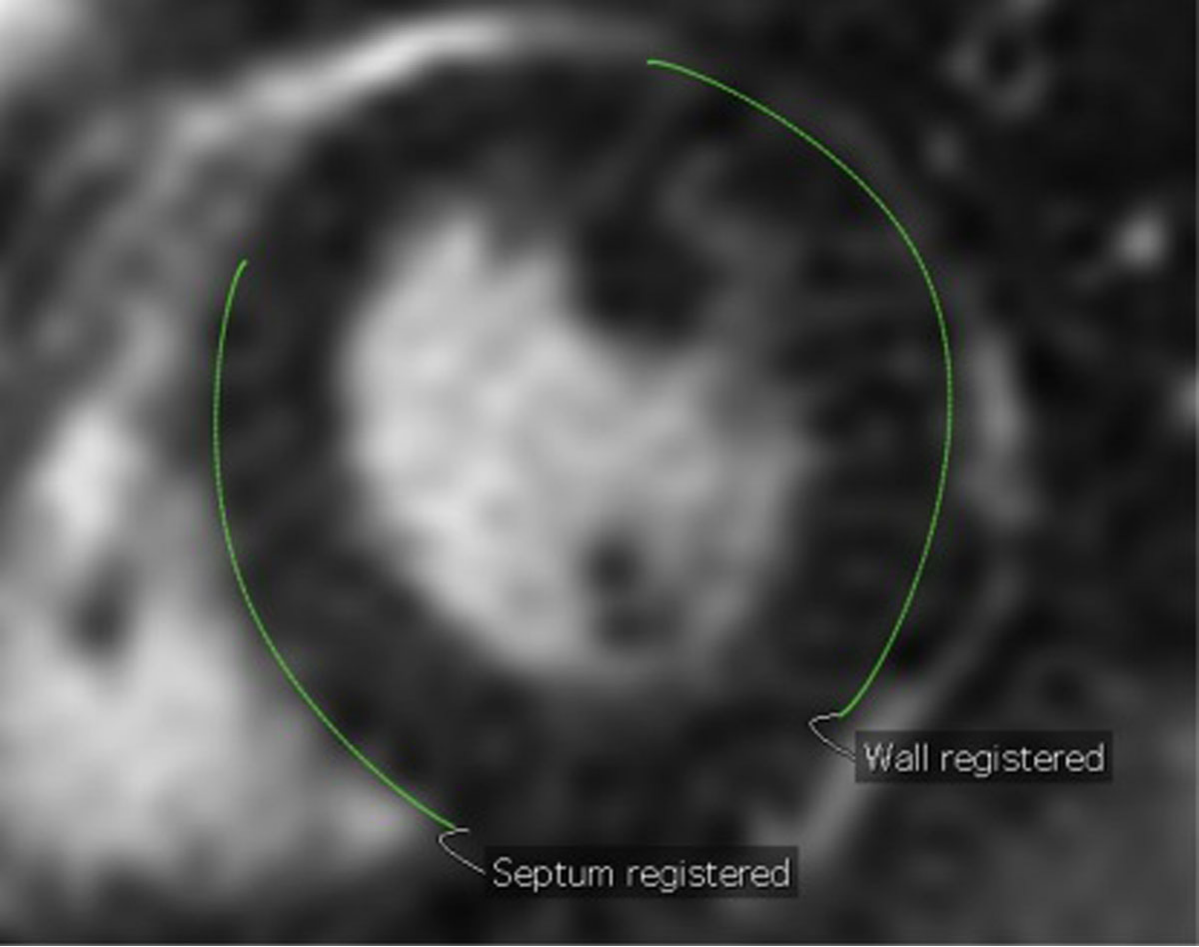
Demonstrating ROIs placed on ventricular free wall and RV septum.

Septal curvature ratio (SCR) at peak systole was analysed in twenty normal controls and twelve patients with diagnosed pulmonary hypertension. All patients underwent a joint cardiac catheterisation and MR procedure. Their pulmonary vascular resistance (PVR) was calculated using phase contrast measures and simultaneous pulmonary arterial (PAP) and capillary wedge pressures. SCR was measured at papillary muscle level in the mid ventricle from kt-SENSE short axis images, using regions of interest applied to the epicardial surfaces of the left ventricular free wall and right ventricular septal wall. [Fig 1] Pearson correlation coefficients of PVR and PAP with SCR were calculated. Sensitivity and specificity of the method were assessed using SCR measures from the control population.

## Results

**Figure 2:**
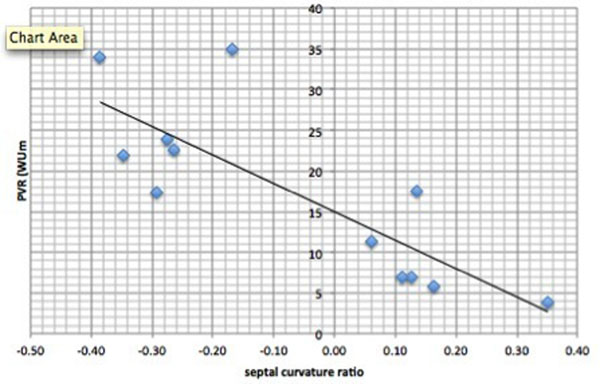
Scatterplot showing the negative correlation between PVR and SCR.

In PH patients, the median SCR was -0.05 (range: -0.39-0.35). PVR was strongly negatively correlated with SCR (r=-0.81, P= <0.05 [Fig 2]), as was the RV-LV pressure difference (r=-0.78, P=<0.05). Median SCR in normal controls was 1.02 (range: 0.9-1.09). Therefore for any threshold between 0.36 and 0.89, SCR had a sensitivity and specificity of 100% for the detection of any level of paediatric pulmonary hypertension. However, diagnostic power was less good for the differentiation of mild and severe PH (specificity 40%, sensitivity 75%).

## Conclusions

This study shows that SCR is a sensitive tool for differentiating normal PVR from those with pulmonary hypertension in the paediatric population. Our approach took advantage of real-time kt-SENSE imaging of the ventricular short axis to reduce apnoea under anaesthesia in MR/catheter procedures. In routine MR, this sequence should make SCR measurement feasible in a wide age range of unsedated children, where scanning must often be rapid. SCR measurement in children is quick to perform and both sensitive and specific at identifying even mild disease. It was also noted that although there were strong correlations between SCR and measures of PAP and PVR, diagnostic differentiation was poor in established disease.

## Funding

BP/VM funded by The British Heart Foundation.

